# Supplementary material for: From sequence to enzyme mechanism using multi-label machine learning
Source: BMC Bioinformatics. 2014 May 19;15:150. doi: 10.1186/1471-2105-15-150 (PMC4229970; doi:10.1186/1471-2105-15-150)
Supplement: Additional file 2 — Java code of ml2db. Additional file ml2db_code.tar.gz contains the Java source code to run the multi-label machine learning experiments and save the results to database. The code’s Javadoc is included. [file 1471-2105-15-150-S2.zip › additional file 2/ml2db/ml2db/doc/mulan/transformations/RemoveAllLabels.html]

RemoveAllLabels


JavaScript is disabled on your browser.


- Overview
- Package
- Class
- Use
- Tree
- Deprecated
- Index
- Help

- Prev Class
- Next Class

- Frames
- No Frames

- All Classes

- Summary:
- Nested |
- Field |
- Constr |
- Method

- Detail:
- Field |
- Constr |
- Method


mulan.transformations

## Class RemoveAllLabels

- java.lang.Object
- - mulan.transformations.RemoveAllLabels

- ---

    

  ```
  public class RemoveAllLabels
  extends java.lang.Object
  ```

  This transformation removes all the label attributes from a
  multi-label dataset

  Version:
  :   2012.02.02

  Author:
  :   Stavros Mpakirtzoglou, Grigorios Tsoumakas

- - ### Constructor Summary

    Constructors

    Constructor and Description

    `RemoveAllLabels()`
  - ### Method Summary

    Methods

    | Modifier and Type | Method and Description |
    | --- | --- |
    | `static weka.core.Instance` | `transformInstance(weka.core.Instance instance, int[] labelIndices)` |
    | `static weka.core.Instances` | `transformInstances(weka.core.Instances dataSet, int[] labelIndices)` Removes the labels from a set of instances |
    | `static weka.core.Instances` | `transformInstances(MultiLabelInstances mlData)` |

    - ### Methods inherited from class java.lang.Object

      `equals, getClass, hashCode, notify, notifyAll, toString, wait, wait, wait`

- - ### Constructor Detail


    - #### RemoveAllLabels

      ```
      public RemoveAllLabels()
      ```
  - ### Method Detail


    - #### transformInstances

      ```
      public static weka.core.Instances transformInstances(MultiLabelInstances mlData)
                                                    throws java.lang.Exception
      ```

      Parameters:
      :   `mlData` -

      Returns:
      :   transformed instances

      Throws:
      :   `java.lang.Exception`


    - #### transformInstances

      ```
      public static weka.core.Instances transformInstances(weka.core.Instances dataSet,
                                           int[] labelIndices)
                                                    throws java.lang.Exception
      ```

      Removes the labels from a set of instances

      Parameters:
      :   `dataSet` - a multi-label dataset
      :   `labelIndices` - the indices of the labels

      Returns:
      :   the transformed dataset

      Throws:
      :   `java.lang.Exception`


    - #### transformInstance

      ```
      public static weka.core.Instance transformInstance(weka.core.Instance instance,
                                         int[] labelIndices)
      ```

      Parameters:
      :   `instance` -
      :   `labelIndices` -

      Returns:
      :   tranformed instance


- Overview
- Package
- Class
- Use
- Tree
- Deprecated
- Index
- Help

- Prev Class
- Next Class

- Frames
- No Frames

- All Classes

- Summary:
- Nested |
- Field |
- Constr |
- Method

- Detail:
- Field |
- Constr |
- Method
